# Circadian rhythm disruption exists in icu patients

**DOI:** 10.1186/2197-425X-3-S1-A428

**Published:** 2015-10-01

**Authors:** K Kiss, I Földesi, B Köves, V Csernus, Z Molnár

**Affiliations:** University of Szeged, Szeged, Hungary; Central Clinical Laboratory, University of Szeged, Szeged, Hungary; Jahn Ferenc Hospital, Budapest, Hungary; Dept. of Anatomy, University of Pécs, Pécs, Hungary; Dept. of Anaesthesiology and Intensive Therapy, University of Szeged, Szeged, Hungary

## Introduction

Circadian rhythms, which are generated by autonomous clock genes called peripheral oscillators, has a pivotal role in harmonising the function of organs and cells of the human body, while disruption of these rhythms (i.e.chronodisruption) is accompanied with a wide spectrum of health disorders. Circadian rhythms in healthy subjects are orchestrated by the dark-time elevation of melatonin (MEL) levels peaking at dawn [1].

## Objectives

The aim of our prospective clinical study was to investigate whether ICU patients have intact circadian rhythm, or they suffer from choronodisruption; and to determine the correlation between MEL and illuminance levels.

## Methods

Mechanically ventilated patients over 18 years, without brain injury (hypoxic, hemorrhage and/or infection) were enrolled into the study in January 2015. Illuminance was measured over the patients' bed by a lux meter and serum MEL levels were determined from blood samples taken from arterial catheters. Measurements took place after admission starting with the closest time point of the regular sampling period of 01:00, 07:00, 13:00 and 19:00 hours with a precision of ± 10 min. for 4 days. Blood samples were kept in total darkness until clotting at room temperature to avoid light induced MEL degradation. The samples were centrifuged for 8 min. at 3000 rpm, then serum samples were placed into -80 °C in 500 µl test tubes dedicated for photosensitive material. For MEL measurement US-CEA908GE ELISA kit was used.

## Results

15 patients were enrolled on a 16 bed multidisciplinary ICU, with age of 63 ± 9 years. Male/female ratio was 9/6. 240 blood samples of 16 patients were analysed for MEL. MEL and illuminance levels are summarised in Table 1. Although illuminance was the lowest at 01:00, but levels were still a lot higher than under physiological circumstances ( < 8 lux) [2]. Furthermore, at 01:00 a MEL peak should occur [1], which was also missing in these patients (Table 1). A repeated correlation analysis between MEL serum concentration and illuminance levels showed a weak within-subject correlation (r=-0.097, P = 0.152), and a stronger, but still non-significant between-subject correlation (r = 0.438, P = 0.103).

## Conclusions

Our results indicate that circadian rhythm is disrupted in ICU patients, as indicated by a) the missing MEL peak during the night; b) by the poor correlation between illumination and MEL levels. Future trials are hence warranted to test whether providing physiological light/dark conditions could resynchronise circadian rhythm and if it has any effect on patient outcomes.

## Grant Acknowledgment

ELISA kits were provided by ACHROS Corp.

## References

[1] Hardeland R, Cardinali DP, Srinivasan V, Warren Spence D, Brown GM (2011). Pandi-Perumal SR. Progress in Neurobiology.

[2] Gabel V, Maire M, Chellappa CF, Reichert SL, Schmidt C, Hommes V (2013). Chronobiol Int.

